# Carapanolides T–X from *Carapa guianensis* (Andiroba) Seeds

**DOI:** 10.3390/molecules201119737

**Published:** 2015-11-24

**Authors:** Teppei Miyake, Sari Ishimoto, Naoko Ishimatsu, Keiichiro Higuchi, Katsuhiko Minoura, Takashi Kikuchi, Takeshi Yamada, Osamu Muraoka, Reiko Tanaka

**Affiliations:** 1 Laboratory of Medicinal Chemistry, Osaka University of Pharmaceutical Sciences, 4-20-1 Nasahara, Takatsuki, Osaka 569-1094, Japan; teppei-727@ezweb.ne.jp (T.M.); saridon55@yahoo.co.jp (S.I.); mini-mini.naoko@hotmail.co.jp (N.I.); rikiaifuni815@yahoo.co.jp (K.H.); minoura@gly.oups.ac.jp (K.M.); t.kikuchi@gly.oups.ac.jp (T.K.); yamada@gly.oups.ac.jp (T.Y.); 2 Laboratory of Pharmaceutical Organic Chemistry, Faculty of Pharmacy Kinki University, 3-4-1 Kowakae, Higashi-osaka, Osaka 577-8502, Japan; muraoka@phar.kindai.ac.jp

**Keywords:** Carapa guianensis, Meliaceae, andiroba, seeds, limonoid, mexicanolide, phragmalin, NO production

## Abstract

Two new mexicanolide-type limonoids, carapanolides T–U (**1**–**2**), and three new phragmalin-type limonoids, carapanolides V–X (**3**–**5**), were isolated from the seeds of *Carapa guianensis* (andiroba). Their structures were determined on the basis of 1D- and 2D-NMR spectroscopy.

## 1. Introduction

Limonoids, a series of structurally-diverse and highly-oxygenated tetranortriterpenes, are mainly found in the Meliaceae family and have been attracting attention from biogenetic and synthetic perspectives [1,2,3]. *Carapa guianensis* Aublet (Meliaceae), known locally as andiroba, is a tall tropical tree that is widely distributed in the Amazonas State of Brazil, and its wood is extensively used as commercial timber. The towering tree of andiroba grows up to 40 m in height. Extracts from its bark, flowers and seeds have been used for centuries by the Amazonian people and exhibit various repellent [4], analgesic [5], anti-bacterial [6], anti-inflammatory [7], wound healing [8], anti-malarial [9], anti-allergic [10] and anti-plasmoidal [11] activities, in addition to acute and subacute toxicities [12]. Our previous study on the components of the seed oil of *Carapa guianasis* revealed the structures of two new unusual 9,10-*seco*-mexicanolide-type limonoids, carapanolides A and B [13], two novel carbon skeletal limonoids, guianolides A and B [14], and carapanolides C–I [15], carapanolides J–L [16] and carapanolides M–S [17]. We herein describe the isolation and structural determination of five novel limonoids, carapanolides T–X (**1**–**5**). The structures of **1**–**5** were determined on the basis of NMR spectroscopy, including 1D and 2D (^1^H, ^1^H-COSY, NOESY, HSQC, HMBC) NMR and HRFABMS.

## 2. Results and Discussion

**Figure 1:**
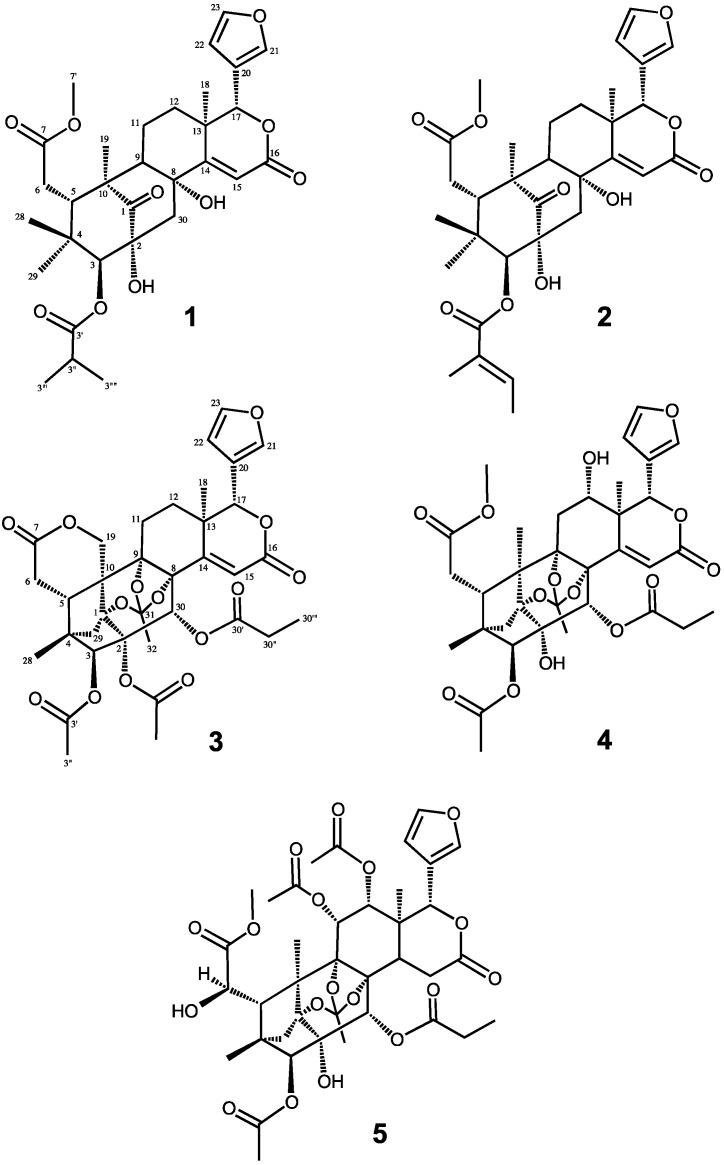
Chemical structures of Compounds **1**–**5**.

Carapanolide T (**1**) (Figure 1) was obtained as a colorless amorphous solid, and its molecular formula was established as C_31_H_40_O_10_ ([M + H]^+^; *m*/*z* 573.2704, calcd. for 573.2697) by HRFABMS, implying 12 on the degrees of unsaturation. IR and UV spectra showed the presence of hydroxylgroups at ν_max_ 3462 cm^−1^, ester groups at ν_max_ 1727 cm^−1^ and αβ-unsaturated δ-lactone at λ_max_ 230 nm (log ε 3.85). ^1^H- and ^13^C-NMR data indicated that eight out of the 12 units of unsaturation came from three carbon-carbon double bonds and three ester carbonyls, including a lactone carbonyl and ketone.

**Figure 2:**
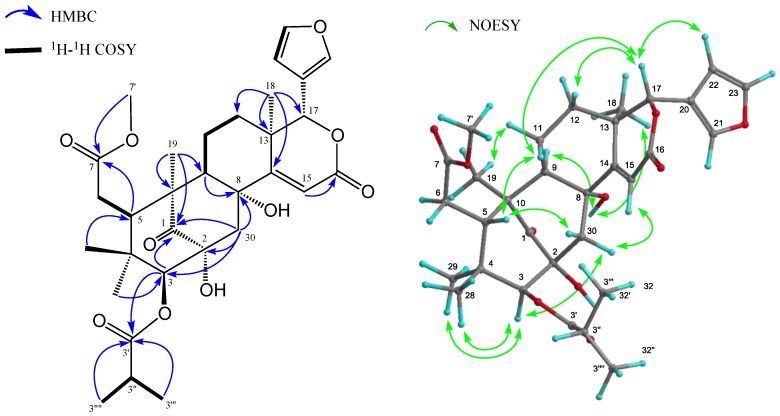
Key HMBC, COSY and NOESY correlations for carapanolide T (**1**).

Therefore, the remaining degrees of unsaturation required **1** to be pentacyclic. The ^1^H- and ^13^C-NMR spectra of **1** (Table 1) indicated the presence of four tertiary methyls (δ_H_ 0.69, 0.86, 1.23, 1.27 (each s)), a 2-methyl propanoyl (δ_H_ 1.25 and 1.27 (each 3H, d), 2.71 (1H, sept); δ_C_ 19.1 and 19.2 (each q), 34.3 (d), 176.2 (s)), methyl ester (δ_H_ 3.71 (s); δ_C_ 52.2 (q), 173.6 (s)), four methylenes (δ_C_ 20.7 (t), 32.9 (t), 33.7 (t), 45.0 (t)), four *sp*^3^ methines, including two oxymethines (δ_H_ 4.80 (s), 5.16 (s)), a furan ring (δ_H_ 6.49 (dd), 7.44 (t), and 7.51 (brs)), an α,β-unsaturated δ-lactone (δ_H_ 5.16 (s), 6.18 (s); δ_C_ 78.8 (d), 116.3 (d), 167.5 (s)), five *sp*^3^ quaternary carbons, including two oxycarbons (δ_C_ 72.8 (s), 76.7 (s)), and a ketone (δ_C_ 216.8 (s)). An analysis of the ^1^H-^1^H COSY spectrum of **1** revealed the partial structures shown in bold face in Figure 2.

In the HMBC spectrum (Figure 2), cross-peaks were observed from Me-18 (δ_H_ 1.27 (s)) to C-12, C-13, C-14 (δ_C_ 164.7 (s)) and C-17 (δ_C_ 78.8 (d)), from Me-19 (δ_H_ 1.23 (s)) to C-1 (δ_C_ 216.8 (s)), C-5, C-9, and C-10, from H-5 (δ_H_ 3.36 (dd)) to C-4, C-6, C-7 (δ_C_ 173.6 (s)) and C-10, from H_2_-30 (δ_H_ 2.51 and 3.55 (each d)) to C-1, C-2 (δ_C_ 76.7 (s)), C-3 (δ_C_ 85.5 (d)), C-8 (δ_C_ 72.8 (s)) and C-9 and from H-15 (δ_H_ 6.18 (s)) to C-8, C-13, C-14 (δ_C_ 164.7 (s)) and C-17. In the ^1^H-^1^H COSY spectrum, (H-5–H_2_-6; H-9–H_2_-11–H_2_-12; H-22–H-23; H-3′′–H-3′′′ and H-3′′′′) revealed the partial structure shown in Figure 2. Significant NOE interactions (Figure 2) were observed from H-3 (δ_H_ 4.80 (s))/Me-28, Me-29; H-5 (δ_H_ 3.36 (dd))/H-11β, Me-28, H-30β; 2-OH (δ_H_ 4.05 (s))/H-12α, Me-19; 8-OH (δ_H_ 2.81 (s))/H-9, H-12α, Me-18; H-17 (δ_H_ 5.16 (s))/H-5, H-11β, H-12β and H-30β, which indicated the α-orientation of H-3, H-9, Me-18, Me-19, 2-OH and 8-OH and the β-orientation of H-5 and H-17. Therefore, the relative structure of **1** was established as shown in Figure 1.

Carapanolide U (**2**) was isolated as colorless needles and shown to have the molecular formula C_32_H_40_O_10_ (*m*/*z*: 585.2701 [M + H]^+^ (calcd for 585.2700) by HRFABMS. IR and UV spectra revealed the presence of hydroxy and ester groups and an αβ-unsaturated δ-lactone at ν_max_ 3537, 1748 and 1719 cm^−1^, and λ_max_ at 230 nm (log ε 4.11). The ^1^H- and ^13^C-NMR spectra of **2** (Table 1) were very similar to those of **1**, except for the absence of the 2-methylpropanoyl group at C-3 and the presence of the tigroyl group at C-3. The relative structure of **2** was determined as shown in Figure 1.

**Figure 3:**
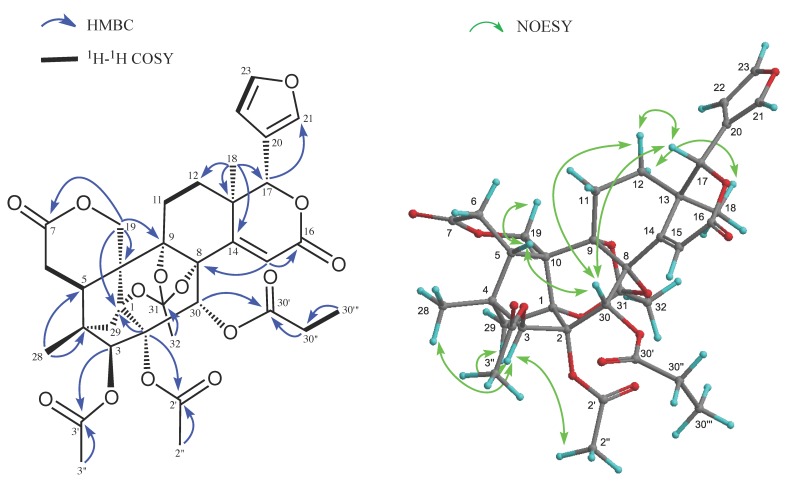
Key HMBC, COSY and NOESY correlations for carapanolide V (**3**).

Carapanolide L (**3**) was obtained as colorless crystals, and its molecular formula was established as C_35_H_38_O_14_ ([M + H]^+^; *m*/*z* 683.2335, calcd. for 683.2340) by HRFABMS, implying 17 on the index of hydrogen deficiency. IR and UV spectra revealed the presence of ester groups and an αβ-unsaturated δ-lactone at ν_max_ 1748 and 1719 cm^−1^ and λ_max_ at 226 nm (log ε 3.73). ^1^H- and ^13^C-NMR data indicated that eight out of the 17 units of unsaturation came from three carbon-carbon double bonds and five ester carbonyls, including two lactone carbonyls. Therefore, the remaining degrees of unsaturation required **3** to be non-acyclic. The ^1^H- and ^13^C-NMR spectra of **3** (Table 2) indicated the presence of two tertiary methyls (δ_H_ 1.02, 1.14 (each s)), two acetyls (δ_H_ 2.04 (s), 2.17 (s); δ_C_ 20.8 (q), 21.8 (q), 169.1 (s), 170.1 (s)), an *n*-propanoyl group (δ_H_ 1.07 (3H, t), 2.25 (1H, m), 2.29 (1H, m); δ_C_ 8.5 (q), 27.4 (t), 173.3 (s)), an orthoacetyl group (δ_H_ 1.68 (3H, s); δ_C_ 20.9 (q), 120.1 (s)), four methylenes, including an oxymethylene (δ_H_ 4.34 and 4.86 (each 1H, d), four *sp*^3^ methines, including three oxymethines (δ_H_ 5.10 (s), 5.27 (s) and 5.78 (s)), a furan ring (δ_H_ 6.44 (dd), .44 (t) and 7.52 (brs)), seven *sp*^3^ quaternary carbons, including four oxycarbons (δ_C_ 82.6, 83.9, 84.2, 84.6 (each s)), and two lactone carbonyl groups (δ_C_ 163.0 and 171.0 (each s)). An analysis of the ^1^H-^1^H COSY spectrum of **3** revealed the partial structures shown in bold face in Figure 3. In the HMBC spectrum (Figure 3), cross-peaks were observed from Me-18 (δ_H_ 1.14 (s)) to C-12, C-13, C-14 (δ_C_ 159.6) and C-17 (δ_C_ 80.4), from Me-28 (δ_H_ 1.02 (s)) to C-3 (δ_C_ 81.3), C-4, C-5 and C-29, from H-30 (δ_H_ 5.78 (s)) to C-1 (δ_C_ 84.6 (s)), C-2 (δ_C_ 84.2 (s)), C-3, C-8 (δ_C_ 82.6 (s)) and C-9 (δ_C_ 83.9 (s)) and from H-15 (δ_H_ 6.05 (s)) to C-8, C-13, C-14 and C-16 (δ_C_ 163.0 (s)). Therefore, the planar structure of **3** was established as phragmalin-1,8,9-orthoacetate [13], and the positions of the two acetyls and an *n*-propyl group were located at C-2, C-3 and C-30, respectively, by detailed ^1^H-^1^H COSY and HMBC correlations (Figure 3). In the NOESY spectrum, significant NOEs (Figure 3) were observed between H-3 and H-29 *pro-S*, H-30 and Me-28, between H-5β (δ_H_ 2.72 (dd)) and H-12β, Me-28 and H-30 between H-15 (δ_H_ 6.05 (s)) and H-17β and H-30, between H-17β (δ_H_ 5.10 (s)) and H-12β, H-15, H-22 and H-30β and between Me-18 (δ_H_ 1.14 (s)) and H-11α, H-12α and Me-32. Therefore, the relative structure of **3** was established as shown in Figure 1.

**Table 1:** ^1^H- (600 MHz) and ^13^C- (150 MHz) NMR spectroscopic data of compounds **1** and **2**.

Position		1			2		
		^1^H ^a^ (*J*, Hz)		^13^C ^b^	^1^H ^a^ (*J*, Hz)		^13^C ^b^
1				216.8			216.7
2				76.7			77.0
3		4.80	s	85.5	4.88	s	85.8
4				39.7			40.0
5		3.36	dd (9.4, 1.5)	41.8	3.39	dd (7.9, 1.1)	41.8
6	α	2.34	dd (17.3, 1.5)	32.9	2.37	m	32.9
	β	2.38	dd (17.3, 9.4)		2.34	m	
7				173.6			173.6
8				72.8			72.9
9		1.85	dd (13.3, 6.0)	60.2	1.87	t (5.6)	60.1
10				47.9			47.8
11	α	1.48	m	20.7	1.67	m	20.7
	β	1.52	m		1.53	m	
12	α	1.28	m	33.7	1.34	m	33.8
	β	2.00	ddd (14.1, 6.8, 3.6)			2.02	m
13				38.5			38.5
14				164.7			164.6
15		6.18	s	116.3	6.16	s	116.3
16				167.5			167.6
17		5.16	s	78.8	5.18	s	79.8
18		1.27	s	23.2	1.28	s	23.2
19		1.23	s	18.3	1.24	s	18.4
20				119.8			119.8
21		7.51	brs	141.7	7.51	br s	141.7
22		6.49	dd (2.1, 0.9)	110.4	6.49	m	110.4
23		7.44	t (2.1)	143.1	7.44	t (1.4)	143.1
28		0.69	s	22.3	0.70	s	22.4
29		0.86	s	22.6	0.88	s	22.6
30	α	2.51	d (16.0)	45.0	2.52	dd (14.9, 1.2)	45.0
	β	3.55	d (16.0)		3.58	d (14.9)	
3′				176.2			167.2
3′′		2.71	sept (6.7)	34.3			128.1
3′′′		1.25	d (6.7)	19.1	6.96	q (7.1)	138.8
3′′′′		1.27	d (6,7)	19.2	1.88	d (7.1)	14.7
3′′′′′					1.92	s	12.4
7'		3.71	s	52.2	3.70	s	52.1
2-OH		4.05	s				
8-OH		2.81	s				

Carapanolide W (**4**) was obtained as colorless crystals, and its molecular formula was established as C_34_H_40_O_14_ ([M + H]^+^; *m*/*z* 673.2492, calcd. for 673.2496) by HRFABMS. IR and UV spectra revealed the presence of hydroxy groups and ester groups and an αβ-unsaturated δ-lactone at ν_max_ 3657, 1728 and 1698 cm^−1^ and λ_max_ at 230 nm (log ε 3.73). The ^1^H- and ^13^C-NMR spectra of **4** (Table 2) indicated the presence of three tertiary methyl groups (δ_H_ 0.74, 1.32, 1.48 (each s)), an acetyl (δ_H_ 2.09 (s); δ_C_ 21.7 (q), 169.1 (s)), an *n*-propanoyl group (δ_H_ 1.16 (3H, t), 2.45 (2H, m); δ_C_ 8.9 (q), 28.1 (t), 173.8 (s)), methyl ester (δ_H_ 3.71 (3H, s); δ_C_ 52.3 (q), 173.9 (s)), three methylene groups, five *sp*^3^ methine groups, including four oxymethine groups (δ_H_ 3.86 (dd), 5.22 (s), 5.35 (s), 5.90 (s)), a furan ring (δ_H_ 6.61 (dd), 7.53 (br s), 7.64 (t)), seven *sp*^3^ quaternary carbons, including four oxycarbons (δ_C_ 83.5 (s), 83.7 (s), 84.2 (s), 86.1 (s)), three ester carbonyls (δ_C_ 169.1 (s), 173.8 (s), 173.9 (s)) and an αβ-unsaturated δ lactone (δ_H_ 6.62 (s), δ_C_ 123.7 (d), 163.5 (s)). Cross-peaks were observed in the HMBC spectrum, from Me-18 (δ_H_ 1.48 (s)) to C-12 (δ_C_ 66.6 (d)), C-13, C-14 (δ_C_ 153.8 (s)) and C-17 (δ_C_ 78.8 (d)), from Me-19 (δ_H_ 1.32 (s)) to C-1 (δ_C_ 84.2 (s)), C-5, C-9 (δ_C_ 86.1(s)) and C-10, from H-15 (δ_H_ 6.62 (s)) to C-8 (δ_C_ 83.7 (s)), C-13, C-14 and C-16 (δ_C_ 163.5 (s)) and from H-30 (δ_H_ 5.35 (s)) to C-1 (δ_C_ 84.2 (s)), C-2 (δ_C_ 83.5 (s)), C-3 (δ_C_ 85.0 (d)), C-8 and C-9. The singlet oxymethine proton at δ_H_ 5.22 was assigned to C-3 through HMBC correlations to C-1, C-2, C-5, C-28, C-29, C-30 (δ_C_ 74.3 (d)) and C-3′ (δ_C_ 169.1 (s)), while another singlet proton at δ_H_ 5.35, showing HMBC correlations to C-1, C-2, C-8, C-9, C-14 (δ_C_ 153.8 (s)) and C-30′ (δ_C_ 173.8 (s)), was assigned to C-30. An oxymethine proton at δ_H_ 5.22 (dd) was assigned to C-12 through HMBC correlations to C-9, C-11, C-13, C-14, C-17 (δ_C_ 78.8 (d)) and C-18. In an analysis of the ^1^H-^1^H COSY spectrum of **4**, H-5–H_2_-6; H_2_-11–H-12; H-22–H-23; H-30′′–H-30′′′) revealed the partial structure. The relative configuration of **4** was established on the basis of NOE interactions. Significant NOE interactions were observed from H-5 (δ_H_ 2.11 (m))/H-11β, H-30, Me-28; H-30 (δ_H_ 5.35 (s))/H-3, H-5, H-15; H-17 (δ_H_ 5.90 (s))/H-5, H-12β, H-15, H-21, H-22, which indicated the α-orientation of H-3, Me-18 and Me-19 and the β-orientation of H-5, H-12, H-17 and H-30. Therefore, the relative structure of **4** was established as shown in Figure 1.

**Table 2:** ^1^H- (600 MHz) and ^13^C- (150 MHz) NMR spectroscopic data of compounds **3**–**5.**

Position		3			4			5		
		^1^H ^a^ (*J*, Hz)		^13^C ^b^	^1^H ^a^ (*J*, Hz)		^13^C ^b^	^1^H ^a^ (*J,* Hz) ^a^		δ_C_^b^
1				84.6			84.2			85.1
2				84.2			83.5			79.6
3		5.27	s	81.3	5.22	s	85.0	4.59	s	83.7
4				46.4			44.6			45.5
5		2.72	dd (4.7, 3.2)	33.8	2.11	m	39.9	3.30	brs	40.1
6	α	2.64	dd (17.0, 3.2)	68.6	2.34	m	33.7	5.98	brs	71.5
	β	2.59	dd (17.0, 4.7)		2.36	m				
7				171.0			173.9			169.4
8				82.6			83.7			84.8
9				83.9			86.1			86.0
10				46.9			48.1			46.2
11	α	2.19	m	25.6	1.97	m	34.6	4.49	d (2.3)	69.4
	β	2.33	m		2.21	dd (14.7, 4.1)				
12	α	1.50	m	26.5	3.86	dd (13.5, 4.1)	66.6	4.48	d (2.3)	71.7
	β	1.64	m							
13				37.6			44.8			38.4
14				159.6			153.8	2.79	dd (10.4, 0.6)	42.3
15		6.05	s	121.0	6.62	s	123.7	2.90	dd (18.7, 10.4)	26.9
								3.22	dd (18.7, 0.6)	
16				163.0			163.5			170.8
17		5.10	s	80.4	5.90	s	78.8	5.98	s	76.9
18		1.14	s	18.7	1.48	s	13.0	1.43	s	15.8
19	α	4.86	d (14.0)	31.4	1.32	s	15.5	1.23	s	13.8
	β	4.34	d (14.0)							
20				119.4			121.4			120.9
21		7.52	br s	141.4	7.53	br s	144.8	7.48	brs	141.0
22		6.44	dd (1.7, 0.6)	109.7	6.61	dd (1.7, 0.9)	109.6	6.46	dd (1.8, 1.5)	110.9
23		7.44	t (1.7)	143.3	7.64	t (1.7)	142.4	7.00	t (1.8)	143.1
28		1.02	s	14.2	0.74	s	14.5	1.10	s	15.4
29	*pro-R*	1.78	d (11.6)	39.2	1.72	d (11.5)	39.8	1.81	d (10.9))	39.9
	*pro-S*	2.38	d (11.6)		1.96	d (11.5)		2.06	d (10.9)	
30		5.78	s	68.1	5.35	s	74.3	6.01	s	69.8
31				120.1			119.7			119.3
32		1.68	s	20.9	1.70	s	16.5	1.76	s	21.1
2′				170.1						
2′′		2.17	s	21.8						
3′				169.1			169.1			169.6
3′′		2.04	s	20.8	2.09	s	21.7	2.18	s	21.2
7′					3.71	s	52.3	3.69	s	53.1
11′										169.7
11′′								2.22	s	21.4
12′										169.6
12′′								1.70	s	20.1
30′				173.3			173.8			172.5
30′′	A	2.25	m	27.4	2.45	m	28.1	2.38	dq (11.2, 7.3)	27.9
	B	2.29	m					2.38	dq (11.2, 7.3)	
30′′′		1.07	t (7.6)	8.5	1.16	t (7.7)	8.9	1.09	t (7.3)	8.6

^a^ Measured at 600 MHz in CDCl_3_. ^b^ Measured at 150 MHz in CDCl_3_.

**Figure 4:**
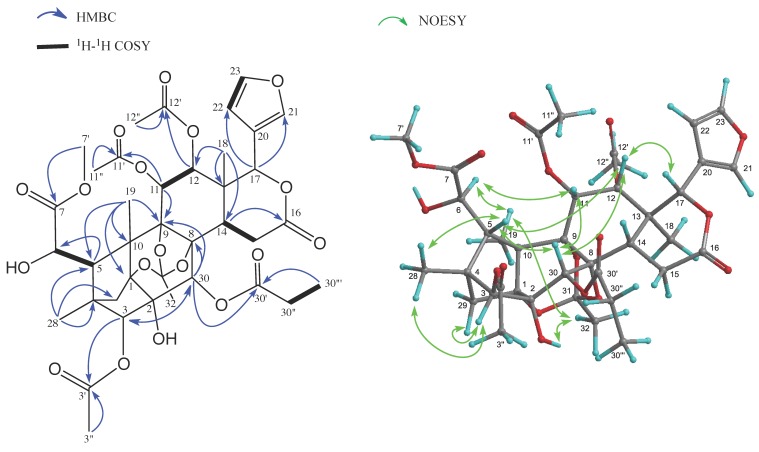
Key HMBC, COSY and NOESY correlations for carapanolide X (**5**).

Carapanolide X (**5**) was isolated as a colorless amorphous solid and had the molecular formula C_38_H_46_O_18_ ([M + H]^+^; *m*/*z* 791.2765, calcd. for 791.2763) as determined by HRFABMS. The IR spectrum showed the presence of a hydroxyl at ν_max_ 3352 cm^−1^ and ester groups at ν_max_ 1742 cm^−1^. ^1^H- and ^13^C-NMR spectra (Table 2) indicated the presence of three methyls (δ_H_ 1.10, 1.23, 1.43 (each 3H, s)), three acetyl groups (δ_H_ 1.70, 2.18, 2.22 (each 3H, s)), a propanoyl group (δ_H_ 1.09 (3H, t), 2.38 (2H, dq), δ_C_ 172.5 (s)), a methoxycarbonyl group ((δ_H_ 3.69 (3H, s), δ_C_ 53.1 (q), 169.4 (s)), *sp*^3^ methylene ((δ_C_ 26.9 (t)), δ-lactone (δ_H_ 5.98 (1H, s), δ_C_ 76.9 (d), 170.8 (s)), a tertiary hydroxyl group (δ_C_ 79.6 (s)), seven *sp*^3^ methines, including five oxymethines (δ_H_ 4.48 (d), 4.49 (d), 4.59 (s), 5.98 (s)), seven *sp*^3^ quaternary carbons, including four oxycarbons (δ_C_ 79.6 (s), 84.8 (s), 85.1 (s), 86.0 (s)), and a furan ring (δ_H_ 6.46 (dd), 7.00 (t), 7.48 (brs)). In the HMBC spectrum, cross-peaks were observed between Me-18 (δ_H_ 1.43 (s)) and C-12 (δ_C_ 71.7 (d)), C-13, C-14 and C-17 (δ_C_ 76.9 (d)), between Me-19 (δ_H_ 1.23 (s)) and C-1 (δ_C_ 85.1 (s)), C-5, C-9 (δ_C_ 86.0 (s)) and C-10, between Me-28 (δ_H_ 1.10 (s)) and C-3 (δ_C_ 83.7 (d)), C-4, C-5 and C-29 and between H-3 (δ_H_ 4.59 (s)) and C-1 and C-2 (δ_C_ 79.6 (s)), C-4, C-5, C-28, C-29, C-30 (δ_C_ 69.8 (d)) and C-3′ (δ_C_ 169.6 (s)), between H-5 (δ_H_ 3.30 (brs)) and C-1, C-3, C-4, C-6 (δ_C_ 71.5 (d)), C-7 (δ_C_ 169.4 (s)), C-10, C-19, C-28 and C-29, between H-6 (δ_H_ 5.98 (brs)) and C-4, C-5, C-7 and C-10 and between H-30 (δ_H_ 6.01 (s)) and C-1, C-2, C-3, C-8 (δ_C_ 84.8 (s)), C-9 and C-30′ (δ_C_ 172.5 (s)). In the ^1^H-^1^H COSY spectrum, (H-5–H-6; H-11–H-12; H-14–H_2_-15; H-22–H-23 and H-30′′–H-30′′′) was observed. Therefore, the positions of two hydroxyls, a propanoyl and a methoxycarbonyl were located at C-2, C-6, C-30 and C-7, while three acetyl groups were located at C-3, C-11 and C-12, respectively (Figure 4). In the NOESY experiments, cross peaks (Figure 4) were observed between H-3 (δ_H_ 4.59 (s)) and H-29 *pro-S*, H-30 and Me-28, between H-5 (δ_H_ 3.30 (brs)) and Me-28 and H-30, between H-6 (δ_H_ 5.98 (brs)) and H-5β and H-11, between H-11 (δ_H_ 4.49 (d)) and H-5β and H-12, between H-12 (δ_H_ 4.48 (d)) and H-5β, H-11 and H-17β, between H-30β (δ_H_ 6.01 (s)) and H-11 and H-12 and between Me-19 and H-6 and Me-32. Therefore, the propanoyl group at C-30 and acetoxy groups at C-11 and C-12 were all α, while the acetoxy group at C-3 had a β orientation. The configuration of C-6 was presumed to be *R*, which was the same as that of carapanolide N [17].

**Figure 5:**
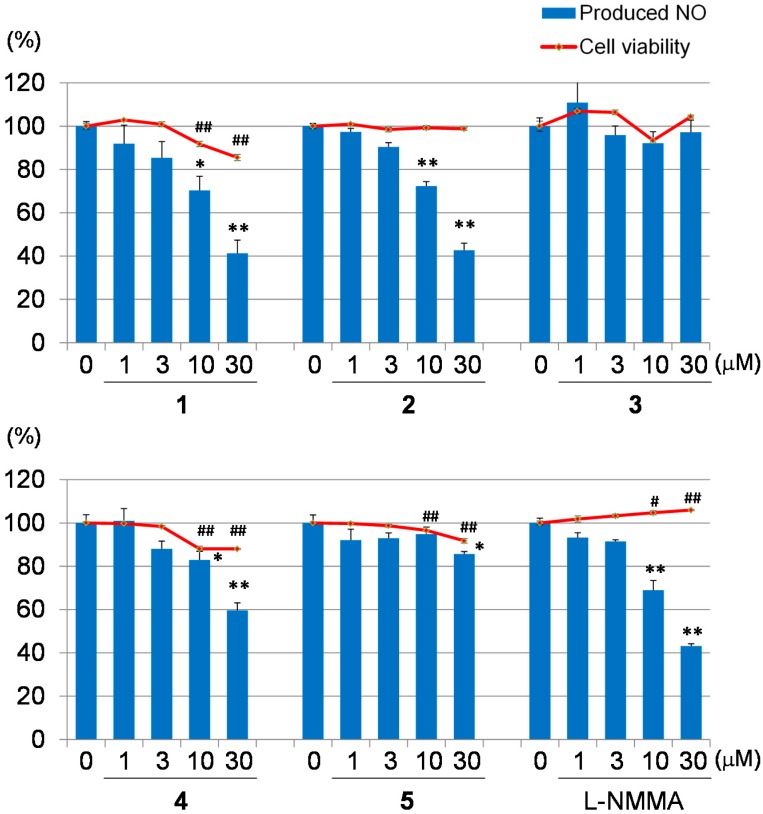
Inhibitory activities on NO production and cytotoxicities of Compounds **1**–**5** and l-NMMA. Each value represents the mean ± the standard error (S.E.) of four determinations. Significant differences from the vehicle control (0 μM) group shown as * *p* < 0.05 and ** *p* < 0.01 in the NO inhibitory assay and # *p* < 0.05 and ## *p* < 0.01 in the cytotoxicity assay.

Macrophages may be a potential therapeutic target for inflammatory diseases [18]. Activated macrophages release pro-inflammatory mediators, such as NO, reactive oxygen, interleukin-1 beta, tumor necrosis factor-alpha and other inflammatory mediators, which play important roles in biological defense. However, the overexpression of these mediators had been implicated in diseases, such as osteoarthritis, rheumatoid arthritis and diabetes, because the increased production of pro-inflammatory mediators has been shown to induce severe or chronic inflammation [18]. In the present study, four limonoids and *N*^G^-monomethyl-l-arginine acetate (l-NMMA), an inducible nitric oxide synthase (iNOS) inhibitor, were evaluated for their inhibitory effects on NO production in LPS-stimulated RAW264.7 cells (Figure 5). To determine safe concentrations, the cytotoxicities of these limonoids against RAW 264.7 were assessed by the MTT assay. In the NO inhibitory assay, Compounds **1** and **2** exhibited comparable NO inhibitory activities (IC_50_ **1**: 22.0 μM; **2**: 23.3 μM) to l-NMMA (IC_50_ 23.9 μM). Of these, **2** did not show cytotoxicities at 1–30 μM. Compound **1** exhibited low cytotoxicity at 30 μM, but not at the effective concentration, namely 10 μM. Compounds **3**–**5** did not exhibit inhibitory effects on macrophage activation (IC_50_ >30 μM). These results suggested that Compounds **1** and **2** have potential as anti-inflammatory disease agents. In a previous study, we revealed inhibitory activities on NO production of a phragmalin-type limonoid, such as carapanolide J (IC_50_ 37.4 μM) [16], and gedunin-type limonoids, such as 7-deacetoxy-7-oxogedunin (IC_50_ 12.8 μM), 6α-acetoxygedunin (IC_50_ 7.9 μM), 6α-hydroxygedunin (IC_50_ 19.1 μM), 6α-acetoxy-7α-deacetoxy-7α-hydroxygedunin (IC_50_ 9.4 μM), gedunin (IC_50_ 4.6 μM) and 7-deacetoxy-7-hydroxygedunin (IC_50_ 8.4 μM), [19]. Compounds **1** and **2** exhibited stronger inhibitory activities on NO production than carapanolide J, however weaker than the gedunin-type limonoids. These data suggest that gedunin-type limonoids are more effective for the inhibition of NO production than phragmalin-type ones in general.

## 3. Experimental Section

### 3.1. General Procedures

Melting points were determined on a Yanagimoto micro-melting point apparatus and were uncorrected. Optical rotations were measured using a JASCO DIP-1000 digital polarimeter. IR spectra were recorded using a Perkin-Elmer 1720X FTIR spectrophotometer (Perkin-Elmer Inc., Wellesley, MA, USA). ^1^H- and ^13^C-NMR spectra were obtained on an Agilent vnmrs 600 spectrometer (Agilent Technologies, Santa Clara, CA, USA) with standard pulse sequences, operating at 600 and 150 MHz, respectively. CDCl_3_ was used as the solvent and TMS as the internal standard. FABMS were recorded on a JEOL-7000 mass spectrometer (JEOL, Tokyo, Japan). Column chromatography was performed over silica gel (70–230 mesh, Merck, Darmstadt, Germany), while medium pressure liquid chromatography (MPLC) was conducted with silica gel (230–400 mesh, Merck). HPLC was run on a JASCO PU-1586 instrument (JASCO, Tokyo, Japan) equipped with a differential refractometer (RI 1531). Fractions obtained from column chromatography were monitored by TLC (silica gel 60 F_254_, Merck).

### 3.2. Plant Material

The oil of (2.03 kg) Carapa guianensis AUBLET (Meliaceae) was collected in the Amazon, Brazil, in March 2013, and was kindly provided by Mr. Akira Yoshino (who is a representative of the “NGO Green Heart Love Amazon Project”). A voucher specimen (CGS-01-2) was deposited in the Herbarium of the Laboratory of Medicinal Chemistry, Osaka University of Pharmaceutical Sciences.

### 3.3. Isolation of Compounds ***1***–***5***

The seed oil of *Carapa guianensis* AUBLET (Meliaceae) (2.03 kg) was dissolved in CHCl_3_, and the CHCl_3_ solution was subjected to CC (silica gel 14 kg), affording 7 fractions: Fraction A (fraction (Fr.) No. 1–85, 1.512 kg) was eluted with *n*-hexane-CHCl_3_ = 1:1; B (Fr. No. 86–179, 229.1 g) was eluted with CHCl_3_; C (Fr. No. 180–20, 29.3 g) was eluted with CHCl_3_–EtOAc = 5:1; D (Fr. No. 221–225, 13.2 g) was eluted with CHCl_3_–EtOAc = 2:1; E (Fr. No. 226–265, 84.5 g) was eluted with CHCl_3_-EtOAc = 2:1; F (Fr. No. 266–290, 25.3 g) was eluted with EtOAc; G (Fr. No. 291–315, 72.8 g) was eluted with EtOAc:MeOH = 5:1; and H (Fr. No. 316–333, 45.4 g) was eluted with MeOH. Residue D was rechromatographed over a silica gel column (CC) (230–400 mesh, 300 g) eluted with *n*-hexane–EtOAc (1:1) to give 13 fractions: D1 (Fr. No. 1–35, 1.52 g), D2 (Fr. No. 36–49, 0.81 g), D(3) (Fr. No. 50–88, 0.70 g), D(4) (Fr. No. 89–115, 0.53 g), D(5) (Fr. No. 116–130, 0.60 g), D(6) (Fr. No. 131–140, 0.52 g), D(7) (Fr. No. 141–205, 0.47 g), D(8) (Fr. No. 206–215, 0.51 g), D(9) (Fr. No. 216–220, 0.42 g), D(10) (Fr. No. 221–240, 0.40 g), D(11) (Fr. No. 241–250, 1.11 g) and D(12) (Fr. No. 251–313, 1.36 g). Fraction D(6) was subjected to CC (230–400 mesh, 40 g) eluted with *n*-hexane–EtOAc (3:1) to give an amorphous solid (24.1 mg) that was separated by HPLC (ODS, 75% MeOH, at 25 °C, flow rate 4.0 mL min^−1^, UV = 220 nm, column 250 × 20 mm i.d., 5 μm) to give Compounds **2** (6.2 mg) and **3** (1.8 mg). Fraction D(8) was subjected to CC (230–400 mesh, 40 g) eluted with *n*-hexane-EtOAc (3:1) to give an amorphous solid (34.0 mg), which was subsequently subjected to CC (230–400 mesh, 40 g) eluted with *n*-hexane–EtOAc (3:1) to give an amorphous solid that was purified by HPLC (ODS, 75% MeOH, at 25 °C, flow rate 4.0 mL min^−1^, UV = 220 nm, column 250 × 20 mm i.d., 5 μm) to give Compounds **1** (7.50 mg) and **4** (3.8 mg). Fraction D(9) was subjected to CC (230–400 mesh, 30 g) eluted with *n*-hexane-EtOAc (3:1) to give an amorphous solid (25.5 mg), which was subsequently separated by HPLC (ODS, 70% MeOH, at 25 °C, flow rate 4.0 mL min^−1^, UV = 220 nm, column 250 × 20 mm i.d., 5 μm) to give Compound **5** (6.3 mg).

### 3.4. Analytical Data

*Compound* **1**: Colorless crystals; mp 83–85 °C; ${\left[\alpha \right]}_{D}^{25}$ +16.6° (*c* 0.1, CHCl_3_); HRFABMS *m*/*z*: 573.2719 [M + H]^+^ (C_31_H_41_O_10_, calcd for 573.2720); UV (EtOH) λ_max_ nm (log ε): 230 (3.85); IR (KBr) ν_max_ cm^−1^; 3462 (OH), 2970, 1727 (O-C=O), 1649 (C=C-C=O), 1461; ^1^H- and ^13^C-NMR, see Table 1. FABMS *m*/*z* (relative intensity (rel. int.)): 573 ([M + H]^+^, 100), 555 (11), 485 (20).

*Compound* **2**: Colorless crystals; mp 133–136 °C; ${\left[\alpha \right]}_{D}^{25}$ −18.1° (*c* 0.1, EtOH); HRFABMS *m*/*z*: 585.2701 [M + H]^+^ (C_32_H_41_O_10_, calcd for 585.2700); UV λ_max_ (EtOH) nm (log ε): 230 (4.11); IR (KBr) ν_max_ cm^−1^: 3537 (OH), 1748, 1719; ^1^H- and ^13^C-NMR, see Table 1. FABMS *m*/*z* (rel. int.): 607 (3) [M + Na]^+^, 585 (6), [M + H]^+^, 567 (6), 485 (7), 83 (100).

*Compound* **3**: Colorless crystals; mp 181–184 °C; ${\left[\alpha \right]}_{D}^{25}$ −74° (*c* 0.4, EtOH); HRFABMS *m*/*z*: 683.2335 [M + H]^+^ (C_35_H_38_O_14_, calcd for 683.2340); UV λ_max_ (EtOH) nm (log ε): 226 (3.73); IR (KBr) ν_max_ cm^−1^: 1748, 1719; ^1^H- and ^13^C-NMR, see Table 1. FABMS *m*/*z* (rel. int.): 683 (100) [M + H]^+^, 641 (5), 586 (7), 507 (7), 95 (21).

*Compound* **4**: Colorless crystals; mp 144–146 °C; ${\left[\alpha \right]}_{D}^{25}$ +43.3° (*c* 0.1, CHCl_3_); HRFABMS *m*/*z*: 673.2492 [M + H]^+^ (C_34_H_41_O_14_, calcd for 673.2496); UV λ_max_ (EtOH) nm (log ε): 230 (3.73); IR (KBr) ν_max_ cm^−1^: 3657 (OH), 1728, 1698; ^1^H- and ^13^C-NMR, see Table 1. FABMS *m*/*z* (rel. int.): 695 (6) [M + Na]^+^, 673 (47) [M + H]^+^, 613 (10), 599 (100).

*Compound* **5**: Colorless amorphous; ${\left[\alpha \right]}_{D}^{25}$ −46.8° (*c* 0.1, CHCl_3_); HRFABMS *m*/*z*: 791.2765 [M + H]^+^ (C_38_H_47_O_18_, calcd for 791.2763); UV λ_max_ (EtOH) nm (log ε): 208 (1.26), IR (KBr) ν_max_ cm^−1^: 3352 (OH), 1742 (O-C=O); ^1^H- and ^13^C-NMR, see Table 2. FABMS *m*/*z* (rel. int.): 791 [M + H]^+^ (55), 735 (5), 95 (54), 43 (100), 329 (14), 176 (47).

### 3.5. Determination of RAW264.7 Cell Proliferation

RAW264.7 cell proliferation was examined according to a method reported previously [20] with few modifications. Briefly, RAW264.7 cells (5 × 10^4^ cells in 100 μL) were seeded onto 96-well microplates and incubated for 24 h. D-MEM (100 μL) containing test samples (final concentration of 100, 30, 10 or 3 μM) dissolved in DMSO (final concentration 0.2%) was added. After the cells had been treated for 24 h, the MTT solution was added. After 3 h of incubation, 20% sodium dodecyl sulfate (SDS) in 0.1 M HCl was added to dissolve the formazan produced by the cells. The absorbance of each well was read at 570 nm using a microplate reader. The optical density of vehicle control cells was assumed to be 100%.

### 3.6. Inhibitory Assay of NO Production

An inhibitory assay of nitric oxide production was performed according to a method reported previously [16] with slight modifications. Briefly, RAW264.7 cells (5 × 10^4^ cells in 100 μL) were seeded onto 96-well microplates and incubated for 24 h. D-MEM (100 μL) containing test samples (final concentration of 100, 30, 10 or 3 μM) dissolved in DMSO (final concentration 0.2%) and LPS (final concentration of 5 μg/mL) were added. After cells had been treated for 24 h, 50 μL of 0.1% *N*-(1-naphtyl)ethylenediamine in H_2_O and 50 μL of 1% sulfanilamide in 5% phosphoric acid were added. After being incubated for 30 min, the absorbance of each well was read at 570 nm using a microplate reader. The optical density of vehicle control cells was assumed to be 100%.

## 4. Conclusions

Two novel mexicanolide-type limonoids, carapanolides T–U (**1**–**2**), as well as three novel phragmalin-type limonoids, carapanolides V–X (**3**–**5**), were isolated from the seeds of *Carapa guianensis* (andiroba). Their structures were determined by spectroscopic analyses. Compounds **1** and **2** were mexicanolide-type limonoids that had OH in C-2 and C-8. Compounds **3**–**5** were phragmalin-type limonoids that were highly oxidized.

In the NO inhibitory assay, Compounds **1** and **2** exhibited similar NO inhibitory activities (IC_50_ **1**: 22.0 μM; **2**: 23.3 μM) against L-NMMA (IC_50_ 23.9 μM). Compound **1** did not exhibit cytotoxicity at 1–30 μM, while Compound **2** exhibited low cytotoxicity at 30 μM, but not at an effective concentration at 10 μM. These results suggest that Compounds **1** and **2** have potential as anti-inflammatory disease agents.
